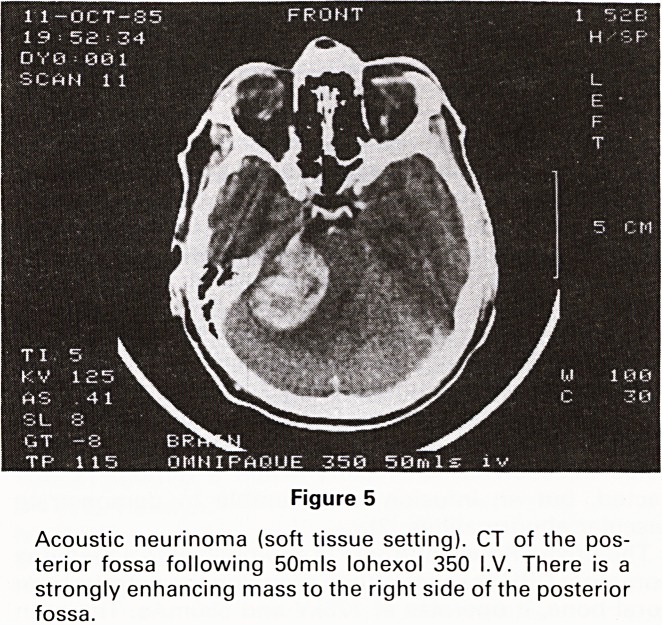# Computed Tomography of the Internal Auditory Canals

**Published:** 1988-02

**Authors:** D. J. Tawn, M. Snow, W. D. Jeans

**Affiliations:** Department of Radiodiagnosis Bristol Royal Infirmary; Department of Radiodiagnosis Bristol Royal Infirmary; Department of Radiodiagnosis Bristol Royal Infirmary


Bristol Medico-Chirurgical Journal Volume 103 (i) February 1988

### Computed Tomography of the Internal Auditory Canals

**D. J. Tawn, M. Snow and W. D. Jeans**
Department of Radiodiagnosis Bristol Royal Infirmary

### INTRODUCTION

The assessment of lesions involving the internal auditory canals and cerebellopontine angle has come to rely heavily on computed tomography (CT). The resolution of modern CT scanners is now comparable to that of complex movement conventional tomography, and CT has the advantage of demonstrating the soft tissue lesion and its intracranial extent, as well as any abnormality of the bone.

The most common reason for radiography and CT scanning of this region is to look for an acoustic neurinoma in patients with sensineural hearing loss. Radiographs of the internal auditory canals should be requested before moving on to CT, since it is unusual to find an abnormality on CT if plain films are normal. Films are normally requested only when there is audiological evidence of sensineural hearing loss.

### PLAIN RADIOGRAPHY

The normal series of films for the internal auditory canals consists of postero-frontal and fronto-occipital (Townes) views, both with a slit diaphragm. These allow simultaneous comparison of both petrous temporal bones. Oblique views (modified Stenvers) are also taken of each petrous temporal bone. These views demonstrate any enlargement or erosion of the internal auditory canal on the affected side. The margins of the internal auditory canal can be seen more clearly using conventional tomography. Complex movement tomography is used, usually hypocycloidal, in order to obtain thin cuts, of the order of 1–2mm, and to abolish linear artefacts. The auditory ossicles are also better seen. However, all these structures are clearly seen on CT, which also demonstrates the soft tissues, so that if an abnormality is demonstrated on plain films CT is the next recommended radiological investigation, if it is available.

### COMPUTED TOMOGRAPHY

As the majority of CT scans of the petrous temoral bone are performed for the investigation of a possible acoustic neurinoma, which enhance strongly with intravenous contrast medium, it is the usual practice to scan after the administration of contrast medium. A non-ionic preparation such as lohexol 350mg iodine/ml or lopamidol 370mg iodine/ml is suitable. A 50ml bolus is given intravenously, 3 to 5 minutes before the scan. Some authors prefer a continuous infusion started five minutes before the scan is commenced (3). It takes several minutes for contrast medium to accumulate in a tumour, but it may remain there for several hours. Therefore a bolus injection is satisfactory when a tumour is suspected, but an infusion is preferable to demonstrate vascular abnormalities (2).

The Bristol Royal Infirmary is equipped with a Siemens Somatom DRH scanner. When scanning the petrous temporal bone, it operates at 125kV and 550mAs. The scan time is 7 seconds and the slice thickness is 2mm. The patient is moved 2mm after each section. Images are taken using both soft tissue and bone window settings, of the same slice. The slices are made in the axial plane, parallel to the anthropological baseline, which extends from the inferior orbital margin through the external auditory meatus. This line is chosen because it avoids direct irradiation of the eyes (13). Scans through the temporal bone are usually sufficient, but the examination is extended to include the entire cranium when indicated.

**Figure 1**

Normal CT of the posterior fossa (bone window settings). The internal auditory canals and lateral semicircular canals can be clearly seen.

In normal studies, the CT scan, at bone settings, shows the shape and size of the internal auditory canal and meatus, while at soft tissue settings it demonstrates CSF in the internal auditory canal, as the subarachnoid space follows the VIIth and VIIIth cranial nerves (Figure 1). Several tributaries of the petrosal vein converge just behind the posterior margin of the internal auditory meatus. In studies using an infusion of contrast medium, this may result in a small area of enhancement at this site (3). This blush is much less likely to be seen following a bolus injection, as the scans are performed well after the peak plasma concentration of contrast medium has passed.

If an unenhanced scan is performed at bone settings the only sign of a tumour may be enlargement and erosion of the internal auditory meatus (Figure 2).

Scanning after IV contrast medium will show tumours extending for 1cm or more into the cerebellopontine angle. This accounts for some 90% of tumours. However, smaller lesions may not be shown, so in patients with a strong clinical indication of VIIth nerve compression and a normal CT scan, air meatography may show smaller and intracanalicular tumours.

### AIR MEATOGRAPHY

After 4–5 mls of air are injected into the subarachnoid

**Figure 2**

CT of the posterior fossa (bone window settings). There is erosion and widening of the right internal auditory canal.

**Figure 3**

Normal CT air meatogram (soft tissue setting). The VIIth and VIIIth nerves can be traced from the brain substance to the apex of the internal auditory canal.

space by lumbar puncture, the patient can be positioned so that the air passes into the cerebellopontine angle and internal auditory canal of the uppermost side. The technique was described by Isherwood, 1972 (6) for use with conventional tomography, but it is even more sensitive when combined with CT (1, 3). The scan is performed in the lateral position, and both sides can be studied in turn with a single injection of air.

With this technique, the facial and vestibulocochlear nerves can be traced from the brainstem to the apex of the internal auditory canal (Figure 3). The anterior inferior cerebellar artery can sometimes be seen looping close to, or even into, the meatus. A possible source of error can occur if a small amount of CSF is trapped at the apex of the canal by air. This gives an area of CSF density at the apex, which may be mistaken for a small neurinoma. The scans must be repeated after a radiologist has tapped the patient's head in an attempt to dislodge the CSF.

### POSSIBLE ARTEFACTS

There are two particular causes of artefact in the posterior fossa. Firstly, linear or 'streak' artefacts are common. These are due to the large difference in attenuation between adjacent bone and soft tissue in this region. Secondly, the inferior margin of the internal meatus may be partially included in the scan, overlapping the subarachnoid space. This may give the impression of an enhancing lesion in the cerebellopontine angle (3).

### DIFFERENTIAL DIAGNOSES

1. Acoustic neurinomas:—These comprise almost 80% of all mass lesions in the cerebellopontine angle (14). The characteristic appearance is of a strongly enhancing lesion, which arises in the internal auditory canal, and erodes the surrounding bone (Figures 4 and 5). The vast majority of acoustic neurinomas enhance (5, 7), whilst when unenhanced nearly 60% are isodense with normal brain tissue (7, 12). Occasionally, localised areas within the tumour do not enhance, but a predominantly non-enhancing acoustic neurinoma is very rare. Bilateral acoustic neurinomas are suggestive of neurofibromatosis (7).
2. Other lesions—Several other lesions occur in the cerebellopontine angle, but these are all relatively rare (11).

**Figure 4**

Acoustic neurinoma (soft tissue setting). CT of the posterior fossa following 50mls Iohexol 350 I.V. An enhancing lesion is seen related to the right internal meatus, which is widened.

**Figure 5**

Acoustic neurinoma (soft tissue setting). CT of the posterior fossa following 50mls Iohexol 350 I.V. There is a strongly enhancing mass to the right side of the posterior fossa.

a. Meningiomas:—These will also enhance after IV contrast medium. They rarely arise in the internal canal, and tend to make an obtuse, rather than acute, angle with the temporal bone. Calcification may be present on the pre-contrast scan and there may be hyperostosis of the temporal bone (3).

b. Facial neurinomas:—These are indistinguishable from acoustic neurinomas when they arise in the internal canal.

c. Trigeminal neurinomas:- These usually arise above the apex of the petrous temporal bone.

d. Epidermoids (Primary cholesteatomas):- The attenuation is close to that of CSF and they do not enhance. The mass effect is important in diagnosis, and air meatography will define the extent.

e. Aneurysms:- Aneurysms of the basilar and anterior inferior cerebellar arteries occur in this region. These will enhance using dynamic CT with an IV infusion of contrast medium.

A few other lesions occur at this site, but they are excessively rare. They include dermoids, plasmacytomas, chordomas and solitary metastasis.

There is resurgence of interest in a theory that tic doloreux and trigeminal neuralgia may be caused by vascular loops coming into contact with the affected nerve. This was first described by Dandy in 1934 (4), and also more recently by other authors (8). Vertebral angiography has been used to demonstrate this (10), and it is feasible that dynamic CT may provide a less invasive alternative method of investigation.

### SUMMARY

CT of the internal auditory canal can now approach the resolution of hypocycloidal tomography, and has the advantage that it will image soft tissue components and intracranial extent.

Small lesions of the internal auditory canal may be demonstrated with air meatography.

### ACKNOWLEDGEMENTS

The authors wish to thank the Department of Medical Illustration for preparing the illustrations, and the Imaging Research Unit for word processing facilities.

### REFERENCES

1. ANDERSON, R., OLSON, J., MERKLE, M., DOWART, R. and SCHAEFFER, S. (1982) CT Air-contrast Scanning of the Internal Auditory Canal. *Ann.Otol.Rhinol.Laryngol.* **91,** 501-505.
2. BURMAN, S. and ROSENBAUM, A. E. (1982) Rationale and Techniques for Intravenous Enhancement in Computed Tomography. *Rad.Clin.N.Amer.* **20,** 15-22.
3. CURTIN, H. D. (1984) CT of Acoustic Neuroma and Other Tumours of the Ear. *Rad.Clin.N.Amer.* **22,** 77-105.
4. DANDY, W. E. (1934) Concerning the Cause of Trigeminal Neuralgia. *Amer.J.Surg.* **24,** 447-456.
5. DUBOIS, P. J., DRAYER, B. P., BANK, W. O., DEEB, Z. L. and ROSENBAUM, A. E. (1978) An Evaluation of Current Diagnostic Radiological Modalities in the Investigation of Acoustic Neurilemmomas. *Radiology* **126,** 173-179.
6. ISHERWOOD, I. (1972) Air Meatography. *Clin.Rad.* **23,** 65-77.
7. JACOBY, C. G., GO, R. T. and BEREN, R. A. (1980) Cranial CT of Neurofibromatosis. *A.J.R.* **135,** 553–557.
8. JANNETTA, P. J. (1980) Neurovascular Compression in Cranial Nerve and Systemic Disease. *Ann.Surg.* **192,** 518-523.
9. KAZNER, E. AULICH, A. and GRUMME, T. H. (1976) Results of Computerised Axial Tomography and Infratentorial Tumors. In *Cranial Computed Tomography* Ed. W. LanKsch and E. Kazner, Springer-Verlag. Berlin, New York.
10. de LANGE, E. E., VIELVOYE, G. J. and VOORMOLEN, J. H. C. (1986) Arterial Compression of the Fifth Cranial Nerve Causing Trigeminal Neuralgia; Angiographic Findings. *Radiology* **158,** 721-727.
11. NAIDICH, T. P. LIN, J. P. LEEDS, N. E. KRICHOFF, I. E. *et al* (1976) Computed Tomography in the Diagnosis of Extra-axial Posterior Fossa Masses Radiology **120**, 333–339.
12. ROBBINS, B. and MARSHALL, W. H. (1978) Computed Tomography of Acoustic Neurinoma. *Radiology* **128,** 367-370.
13. SHAFFER, K. A., HAUGHTON, V. M. and WILSON, C. R. (1980) High Resolution Computed Tomography of the Temporal Bone. *Radiology* **134**, 409-414.
14. TAYLOR, S. (1982) The Petrous Temporal Bone (Including the Cerebellopontine Angle). *Rad.Clin.N.Amer.* **20,(1)** 15-22.

## Figures and Tables

**Figure 1 f1:**
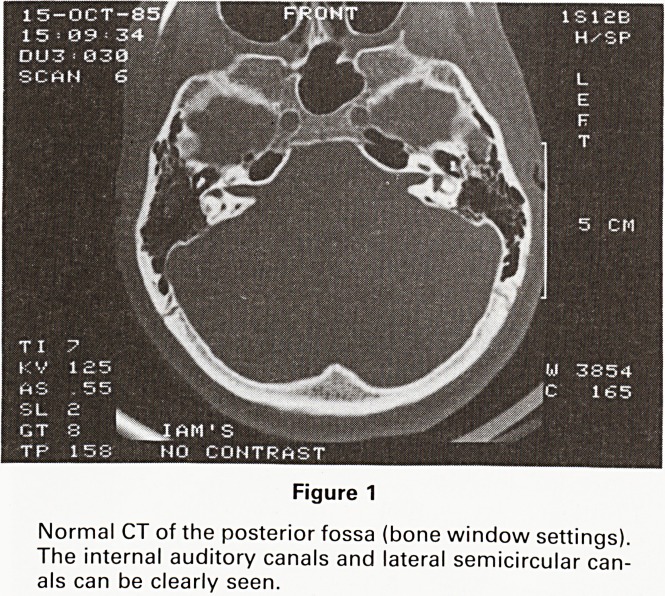


**Figure 2 f2:**
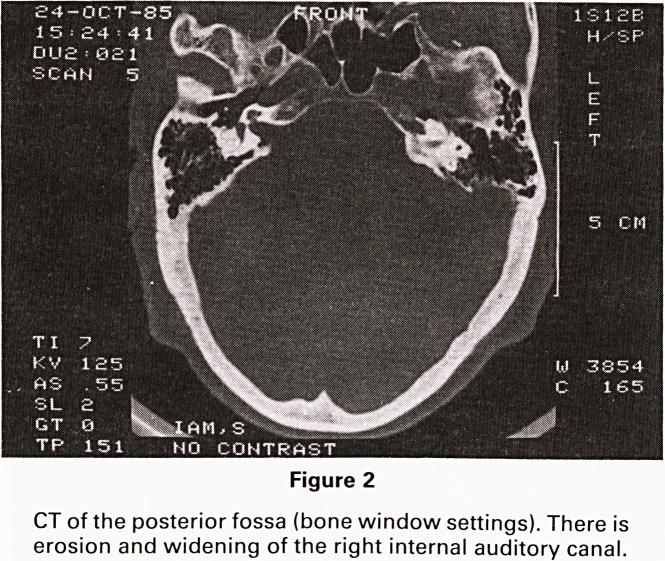


**Figure 3 f3:**
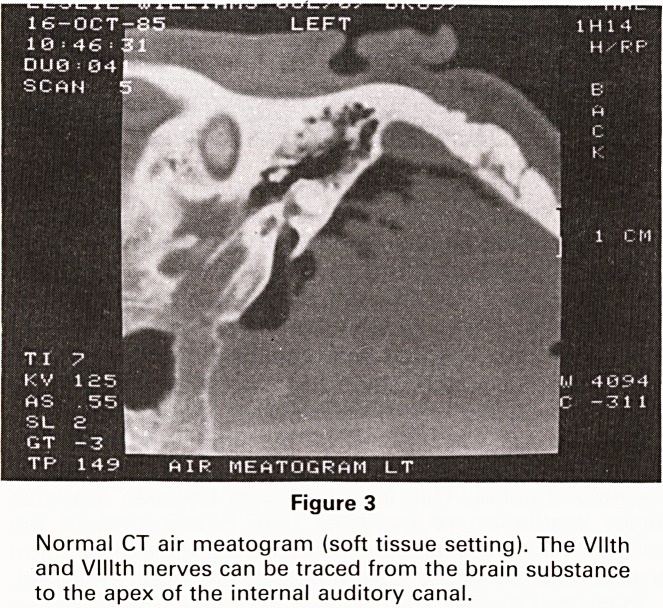


**Figure 4 f4:**
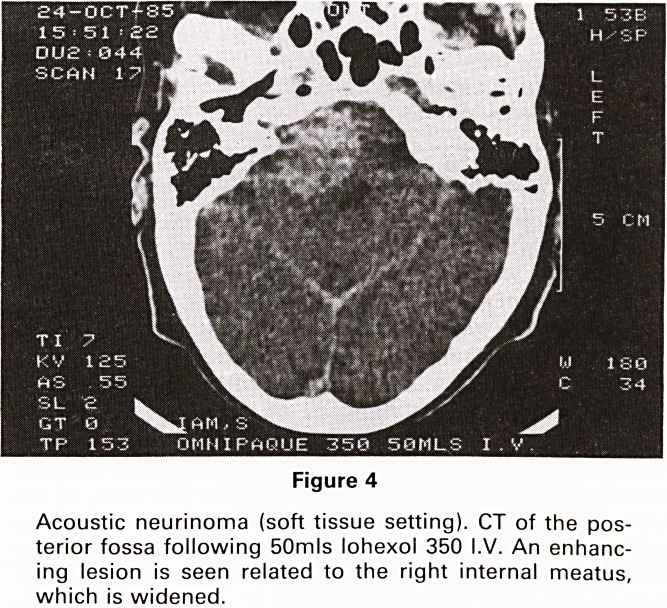


**Figure 5 f5:**